# Still counting sperm? Why novel, truly informative measurements of testis function in male infertility are urgently needed

**DOI:** 10.1007/s12020-025-04453-y

**Published:** 2025-10-11

**Authors:** Chiara Furini, Francesco Costantino, Antonio RM Granata, Giorgia Spaggiari, Daniele Santi, Manuela Simoni

**Affiliations:** 1 Unit of Endocrinology, Department of Medical Specialties, Azienda Ospedaliero- Universitaria of Modena, Modena, Italy; 2 Department of Biomedical, Metabolic and Neural Sciences, University of Modena and Reggio Emilia, Modena, Italy; 3 Unit of Andrology of the Unit of Endocrinology, Department of Medical Specialties, Azienda Ospedaliero-Universitaria of Modena, Modena, Italy

**Keywords:** Couple infertility, Male infertility, Semen, Couple, Assisted reproduction

## Abstract

Couple infertility is estimated to affect between 13% and 18% of all couples of reproductive age, with male factors accounting for at least 50% of cases. Semen analysis is the first-line investigation for all male partners of couples referred for fertility evaluation. However, semen analysis is far from being a good predictor of the ultimate outcome: pregnancy. For instance, in approximately 25% of infertility cases, conventional semen parameters are considered ‘normal’, leading to a diagnosis of so-called ‘unexplained infertility’. Nonetheless, this tool is central in the assessment of male fertility potential. This review first provides a historical perspective on the evaluation of male infertility based on semen analysis, illustrating the evolution of diagnostic approaches over time. The second section explores major changes in the interpretation of semen analysis and highlights the need for alternative methodologies. Finally, the last section examines critically the limitations and pitfalls of semen analysis in the diagnostic workup of male infertility and suggests exploring radical new approaches. The aim of this work is to raise awareness of the clinical limitations of conventional semen analysis and, consequently, to emphasize the urgency of identifying new strategies for diagnosing male infertility and optimizing treatment decisions both a priori and during potential interventions.

## Introduction

Infertility is defined as the inability of a couple to achieve pregnancy after at least 12 months of regular sexual intercourses without contraception [1–3]. Couple infertility is estimated to affect from 13% to 18% of all couples in reproductive age, globally [4]. Both partners could contribute to couple infertility and male factor is estimated to account for at least 50% of all cases [5]. Thus, couple infertility should not be attributed only to the female partner and the couple must be evaluated with a detailed diagnostic work-up with a joint collaboration between gynecologists and andrologists. Many guidelines and position statements, produced by different scientific societies, provide details about the diagnostic work-up of the male partner of an infertile couple [2, 6, 7]. Although these guidelines differ considering several suggestions/recommendations, they share the primary role assigned to semen analysis, which is still the gold standard approach to evaluate male fertility. This analysis assesses semen quantity and quality by evaluating the number, morphology, and motility of spermatozoa as markers of fertility potential [8–10]. To access this information, the World Health Organization (WHO) produced a manual to assist biologists performing the analysis, as well as clinicians interpreting the results.

**Fig. 1:**
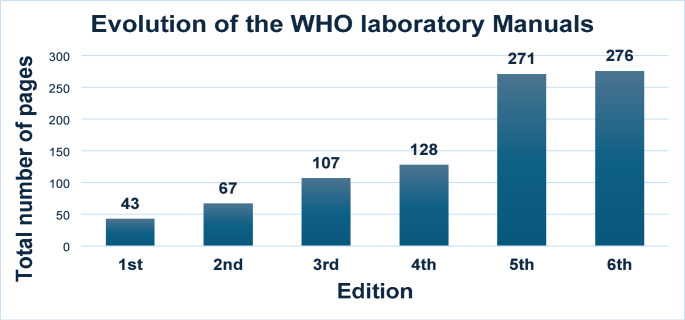
The evolution in pages of the World Health Organization (WHO) laboratory Manual according to subsequent editions

The sixth edition of the WHO manual was published in 2021 [11]. Compared to the first edition from 1980, the latest versions provide more and more detailed guidance, as reflected in its increased number of pages (Fig. 1). However, does this growing body of reccomendations on semen analysis translate into greater prognostic accuracy? This paper reviews strengths and limitations of semen analysis in the evaluation of male fertility potential and challenges the role of the WHO manual in clinical practice.

## The history of semen analysis evaluation

Semen analysis became a part of clinical practice only at the end of the 20th century, following the first edition of the WHO manual published in 1980 [12]. Before the discovery of spermatozoa, male reproductive capacity was evaluated by identifying anatomical defects during physical examination and/or observable signs and symptoms such as testicular pain, sexual dysfunction, or changes in ejaculate volume.

The earliest attempts to investigate human conception and infertility can be traced back to ancient texts, particularly from practitioners in ancient Greece and Rome, such as Democritus, Alcmaeon, Hippon, Empedocles, Hippocrates, Aristotle, Soranus, and Galen. Since infertility in Greek and Roman societies was often regarded as a source of humiliation particularly for women, the wise men of the time were also consulted to address fertility issues. In this context, Aristotle devised an empirical test to evaluate the quality of semen. His ‘water test’ was based on the assumption that fertile men produce hotter semen that would sink in water, while infertile semen would float [13]. Evidence from other ancient societies, such as the Sumerians, Akkadians, and Egyptians, shows theoretical models of conception based on the interaction of male and female ‘seeds’, with semen playing a crucial role. Although these ancient concepts were hypothetical and unproven, they remained influential for centuries until the discovery of sperm and eggs in the 17th and 19th centuries, respectively [12].

In the mid-17th century, Antoni van Leeuwenhoek developed the first microscope that enabled Johan Ham, a student at the Medical School of Leyden, to discover spermatozoa. Ham observed male gametes in the semen of a man suffering from gonorrhoea, referring to them as ‘animalcules spermatiques’ [12]. In 1677, Ham’s microscopic findings reported detailed seminal liquefaction, the prevalence of live sperm cells (which did not survive beyond 24 hours), their motility patterns, and the characteristics of both their heads and tails [14]. This represented a major breakthrough in reproductive medicine, eventually leading to the development of the postcoital or Sims-Huhner test in 1866 [15]. This test, used in infertility investigations, assessed the characteristics of female cervical mucus (i.e. amount, viscosity, elastic quality, and cellularity) after a sexual intercourse, as well as the presence and quantity of motile spermatozoa within it. However, despite its widespread use, the Sims-Huhner test never reached scientific validity and showed low accuracy, since it was not able to identify the sufficient number of active spermatozoa in the cervix required to predict a pregnancy [16]. In the context of male fertility diagnostics, these discoveries laid the groundwork for the semen analysis methods used today [12].

All these advancements in knowledge culminated in 1980 when the WHO began collaborating with scientists to establish standards for high-quality semen analysis and develop a proper manual [12]. These efforts began in 1973 with the creation of the Task Force on Methods for the Regulation of Male Fertility (Male Task Force), whose primary goal was to develop new contraceptives for men. To achieve effective contraception, the Male Task Force required standardized parameters capable of evaluating the contraceptive efficacy of new compounds [17–19]. It is curious and worth mentioning that the manual currently used to study male infertility was originally created with a completely opposite intent, namely male contraception. With these initial steps, the WHO published the first edition of the manual, which, as previously mentioned, has been further refined and expanded over the following 41 years.

### Semen analysis: clinical utility

Conventional semen analysis is the core investigation in fertility assessments, but its ability to predict pregnancy is limited and nuanced. Indeed, semen analysis parameters have some association with pregnancy outcomes, but their predictive power is generally weak and inconsistent. Several systematic reviews and large cohort studies have been conducted so far, but no one was really useful to identify a clear threshold values able to predict pregnancy achievement [20–22]. In fact, routine semen analysis cannot reliably predict the chance of pregnancy or differentiate fertile from infertile men except in extreme cases [22]. In addition, semen analysis gives no information about spermatogenesis within the testis. Thus, the clinical utility of conventional semen analysis is limited. From a clinical point of view, the semen analysis should assess a man’s capacity to produce sperm of sufficient quantity and quality, defined as the ability to reach and fertilize the oocyte. While evaluating sperm count may be considered a relatively straightforward step, the most significant challenge for diagnostic tools lies in assessing sperm quality, referred here as ‘sperm competence’. The ability of conventional semen analysis to assess sperm competence remains questionable.

To overcome this inherent limitations of semen analysis and for the sake of standardization, the WHO manuals were progressively modified. First, WHO introduced more detailed evaluations of parameters such as sperm motility and normal morphology. The assessment of sperm motility has undergone several modifications along WHO manual editions, with a even more detailed distinction between different types of movement, recognizing the clinical significance of these differences. This new classification introduced more challenges in semen evaluation because it is very difficult for the operator to visually distinguish between the different types of sperm motility. Moreover, this detailed motility description does not correlate with a real fertilizing ability. Similarly, the definition and assessment of sperm morphology, particularly the classification of typical or normal forms, evolved across the editions of the WHO manual editions but it is still largely based on the assumption that “nice is good” (the καλὸς καὶ ἀγαθός principle of the ancient Greeks), while the experience with assisted reproduction technologies (ART) demonstrates that also ugly sperm can produce embryos. In the first edition, the evaluation of sperm morphology was relatively basic, focusing on general descriptions of sperm structure. Since the third edition, WHO introduced a significant advancement with the introduction of strict criteria for sperm morphology assessment, emphasizing the importance of quality control and standardization in morphology assessment [18]. However, also this parameter is still unable to really predict the sperm competence and it is not really useful clinically [23]. Second, WHO incorporated additional “second-level” examinations to provide more specific insights into sperm quality. Only the fifth manual edition introduced a turning point by acknowledging the need for additional tests to provide a more comprehensive assessment of male fertility, which was further developed in the last edition. However, they are not routinely applied due to their inherent costs and requirements, as well as their continued inability to accurately predict male fertility potential.

As a result of this attempt to improve semen analysis accuracy, the length of the WHO manual has progressively increased (Fig. 1). However, the last WHO manual edition emphasizes that, while semen analysis can offer insights into male fertility potential, it does not distinguish between fertile and infertile men, thereby shifting away from the previous concept of ‘reference range’ to ‘decision limits’ [11]. The 5th percentile of semen parameters, calculated on the distribution of semen samples of fertile men, no longer serves as a cut-off value between fertility and infertility, since semen parameters per se resulted unable to resolve this dichotomy [11]. Therefore, making semen analysis more and more complicated over the years did not improve much the ability to understand what’s going on within the testis, which is essential to make a correct diagnosis and set up the proper therapy. In this sense, and differently from all other fields in medicine, in andrology we still rely on a rather “primitive” and often uninformative diagnostic tool.

## How the advent of assisted reproduction technologies (ART) changed the clinical role of semen analysis

ART is defined as any fertility-related treatment involving the manipulation of gametes and/or embryos [24]. The advent of ART provided a significant chance for infertile couples to achieve pregnancy. The introduction of intra-cytoplasmic sperm injection (ICSI) in 1990s further improved the efficacy of ART [25], reducing the emphasis on evaluating male fertility [26]. Indeed, ICSI involves the direct injection of a single spermatozoon into an oocyte, bypassing the preliminary penetration steps required in natural fertilization or in in vitro fertilization (IVF). Therefore, only a few sperm are needed to achieve pregnancy with ICSI, reducing the need for extensive sperm quality assessment. Prior to ICSI, detailed analysis of sperm count, motility and morphology was essential to determine the feasibility of conventional ART based on intrauterine insemination (IUI) and IVF. With ICSI, even semen with suboptimal characteristics can result in successful fertilization [27, 28], further demonstrating the scarce utility of meticulously assessing parameters like morphology and motility.

Today, ICSI stands as the prevailing laboratory technique in ART, accounting for nearly 70% of all cycles [29]. This approach allows refractory cases to achieve pregnancy and obtain delivery rates similar to those of IUI and IVF with apparently normal gametes [30]. As a consequence, although male factors account for half of infertility cases in couples, the male partner is often regarded merely as the ‘sperm provider’. This perception has significant consequences, such as the idea that there is no clinical need to identify tools more accurate than semen analysis to evaluate male fertility. This is, however, the limited vision of the gynecologists performing ART, since it became clear over the years that addressing male factor carries pivotal benefits: firstly, it increases the chances of obtaining a pregnancy, natural and after ART. Secondly, it can ease and reduce the impact of the medical procedures women must undergo [31]. Finally, male infertility is currently recognized as a proxy of general health and assessing testicular spermatogenesis, one of the processes in the body with the highest cellular turnover, may provide insights into overall fitness and physical well-being [32].

## Semen analysis pitfalls

The inability of conventional semen analysis to distinguish between infertile and fertile individuals derives from different factors. First, semen parameters can vary significantly from day to day in the same individual [33]. Consequently, the WHO and male infertility guidelines suggest examining at least two semen samples to define more accurately a proxy of male fertility potential [1–3, 11]. A study published by Mallidis and colleagues in 1991, aimed to assess the proportion of within-subject and between-subject variation in seminal parameters, evaluated sperm parameters in 673 semen samples collected from seven healthy men participating in a donor insemination program [33]. Interestingly, the largest proportion of overall variance attributed to within-subject differences ranged from a minimum of 54% for sperm concentration to a maximum of 96% for sperm motility [33]. Similarly, a more recent retrospective cohort study involving 5,240 male partners of infertile couples confirmed significant within-subject variability in semen volume, sperm concentration, motility and morphology and total sperm count, with variability ranging from 28% to 34% [34]. Moreover, in this setting the inter-operator variability is extremely relevant. Thus, the WHO manual stresses the role of both quality control and quality assurance processes at ensuring reproducible results and standardization. These steps serve as critical checks and balances across all stages—pre-analytical, analytical, and post-analytical [35]. However, to date, data on the clinical reliability of these steps in enhancing the fertility prediction of semen analyses remain inconclusive [36–38].

In spite of quality control, even in the presence of optimal accuracy, the ability of semen parameters to predict pregnancy is close to zero. Several prospective studies of men attempting conception revealed positive associations between conventional sperm parameters (i.e. total sperm count, concentration, motility and morphology) and fertility markers such as time to pregnancy, likelihood of spontaneous conception, and ART outcomes [9, 39, 40]. So far, however, should a direct relationship between semen parameters and pregnancy potential exist, a threshold able to predict a ‘normal’ fertility status has not been identified.

Masturbation is the recommended method for semen collection [11]. However, growing concerns are emerging about its reliability. Several studies have shown that semen samples collected after coitus using a special collection device are superior to those obtained through masturbation, particularly in terms of semen volume, sperm concentration, morphology and motility [41–43]. However, these devices are neither routinely used, nor considered in clinical practice. It has been suggested, and widely accepted, that sexual arousal during sexual intercourse reaches higher levels, leading to a more intense orgasm and possibly a better-quality ejaculate, compared to masturbation [44–46]. Some researchers investigated how sexual arousal and orgasm intensity might contribute to the within-subject variations in semen quality during masturbation [47]. In 1996, Van Roijen and colleagues found no differences in semen characteristics between samples obtained with or without visual erotic stimulation in a small cohort of healthy donors and subfertile men [48]. In contrast, a more recent prospective study reported a consistent increase in semen volume and total motile sperm count after three days of sexual stimulation using video broadcasts [49]. Thus, the data of the literature remain comprehensively inconclusive. Accordingly, the WHO sought to standardize semen collection by recommending that it is obtained through masturbation after a minimum of two days and a maximum of seven days of ejaculatory abstinence, in an attempt to ensure consistency in the collection process [11].

Finally, high heterogeneity among laboratories performing semen analysis exists *de facto*. Probably because of the intrinsic, above mentioned limitations, and of a plethora of unnecessarily complicated methodologies, consequence of a sacred zeal for standardizing parameters that are difficult to objectify, a surprisingly large number of laboratories do not fully adhere to WHO guidelines for semen analysis. It has been reported that only 60% of United States laboratories comply with WHO standards, with even lower adherence rates reported in the UK, where only 5% of laboratories follow these guidelines [50, 51]. Obviously, the increasing complexity of the WHO manuals for the poor performance does not motivate toward strict standardization. This expansion not only covers additional semen tests, but also includes more detailed guidance on conventional analysis. For this reason, the last three editions of the WHO manual (1999, 2010, 2021) devote an entire chapter to quality assurance and both external and internal quality control, correclty emphasizing their crucial role in reducing variability within and between andrology laboratories and increasing analytical accuracy. Unfortunately, not always analytic accuracy corresponds to diagnostic and prognostic accuracy.

The WHO effort toward standardization and detailed interpretation of semen analysis, however, are not in vain, and are essential for research purposes, where researchers must use a common framework to obtain reliable and comparable findings. In 2022, Björndahl and colleagues highlighted this issue asserting that “when methods with a high degree of uncertainty are used, differences between normal and pathological conditions are likely to be impossible to discover” [52]. Therefore, the authors proposed a checklist for peer-reviewed journals which researchers should complete and submit with their manuscript [52]. This effort is necessary to minimize potential biases arising from the inherent limitations of semen analysis in research applications, ensuring the generation of strong and reliable results.

## Semen analysis as a marker of male infertility treatment efficacy: when can a treatment for male infertility be considered effective?

The limitations of semen analysis negatively impact both the diagnostic and the therapeutic management of male infertility. Indeed, any treatment used for the male partner of an infertile couple requires a clear outcome to assess its effectiveness. While pregnancy is the ultimate goal, its use as an endpoint is controversial. First, achieving pregnancy depends on both partners, making it an indirect measure of male fertility. Second, evaluating treatment effects on the gonads and improvements in sperm parameters provides a more measurable and directly relevant endpoint for the treated individual [53]. For this reason, improving seminal parameters has been proposed as the primary objective of therapy. However, if semen analysis is not accurate enough in diagnosing male infertility, how can it reliably assess treatment efficacy? Powerful biomarkers able to identify individuals who genuinely benefit from a drug or surgical procedure for male infertility are missing.

Historically, a doubling of sperm concentration from baseline has been suggested as a criterion to identify idiopathic oligozoospermic men who respond adequately to FSH administration [54]. More recently, analysis of real-world data has demonstrated that conventional semen parameters can predict pregnancy outcomes in idiopathic infertile men treated with FSH 150 IU three times per week. This study also identified a mathematical function capable of discriminating between successful and unsuccessful fathering based on the difference between baseline and treatment values in sperm concentration [55]. On the other hand, other cohorts analysed highlighted no significant increase in total sperm count after treatment, reinforcing the idea that conventional semen analysis alone is insufficient to understand sperm quality [53]. Thus, combining these evidences with the pitfalls intrinsic to semen analysis, it is clear that new specific marker of male fertility potential must be identified.

## Which examinations could improve the semen analysis diagnostic power?

Semen analysis may include both basic and advanced investigations. Adavanced tools entered the second-level examinations described by the WHO manual. These techniques include sperm DNA fragmentation index (sDF), antisperm antibody testing, genetic testing, and computer-assisted semen analysis.

In human reproduction, DNA integrity is crucial in germ cells, not only for cell survival but also for the transmission of genetic information to the offspring. Both physiological and pathological, endogenous and exogenous, factors can impair DNA integrity during sperm maturation and storage in the epididymis [56]. Given that DNA integrity is essential for embryo development and implantation [57], the latest WHO manual acknowledges four tests to assess sDF: (i) the TUNEL assay (terminal deoxynucleotidyl transferase Dutp nick and labelling), (ii) the sperm chromatin dispersion assay, (iii) the acridine orange flow cytometry assay, and (iv) the Comet assay [11]. In infertile men, sDF is significantly increased, regardless of the sDF measurement method used [53]. The sixth edition of the WHO manual highlights that assessing sperm chromatin quality “could represent an important addition in the work-up of male infertility” and may serve as a promising biomarker for identifying infertile men [11]. Nevertheless, several limitations still prevent sDF testing from being widely adopted in routine clinical practice. First, there is no universally accepted cutoff value: proposed thresholds vary depending on the assay used, the patient population, and the clinical setting, with some evidence suggesting that levels < 15% may predict better outcomes in women under 40 years [58, 59]. Second, large, prospective randomized controlled trials are still missing, making it difficult to demonstrate how SDF testing can directly influence management and improve live birth rates. Third, methodological heterogeneity remains a major issue: different assays measure different types of DNA damage, show variable reproducibility, and lack standardized laboratory protocols [59]. Finally, practical barriers such as cost, equipment availability, and the need for technical expertise further limit routine implementation. Until these challenges are addressed, sDF testing is likely to remain a useful adjunct in selected cases rather than a universally applied diagnostic tool.

Another relevant second-level examination is the use of fluorescent in situ hybridization (FISH) testing for sperm aneuploidy, which identifies genetic abnormalities that may contribute to fertilization or implantation failure [60]. FISH probes typically target chromosomes 13, 18, 21, X, and Y, as these are associated with numerical anomalies compatible with life. While commercial probes for other chromosomes and genetic *loci* exist, they are expensive and not used routinely, typically reserved for selected cases such as couples with a history of recurrent miscarriages [61].

Among the methods suggested to improve semen analysis accuracy and reliability, the Computer-Assisted Semen Analysis (CASA) systems have been developed. The main aim of these tools is to objectively assess sperm concentration, motility, and morphology, using digital imaging and software algorithms to reduce subjectivity and improve the consistency and accuracy of results compared to manual methods. Indeed, among the challenges in semen analysis accuracy, inter and intra-technician variability of this examination remain high. Thus, since the 1980s, CASA technology has been increasingly suggested in both research and clinical practice to substitute manual semen analysis [62]. Evidence in the literature suggests that CASA systems are a valid alternative for evaluating semen parameters, particularly when it comes to sperm concentration and motility measurements [63, 64]. Moreover, CASA has automated certain aspects of conventional semen analysis, allowing laboratories to process a greater number of samples more efficiently than manual methods. However, some studies suggested the limited usefulness of this analysis, demonstrating significant differences in all semen parameters measured with CASA and manually, avoiding a clear recognition of which one is the most accurate approach [65, 66]. It should be considered that the poor technological advancement of CASA systems can be attributed, at least in part, to the resistance of andrologists to introduce this innovation into the routine practice, which demotivated commercial entities to develop and refine automated semen analysis platforms. This scenario seems to be in contrast to other domains of laboratory medicine, where automation has led to transformative improvements in diagnostic accuracy, workflow efficiency, and personnel safety. For example, clinical microbiology has benefited substantially from automation. Automated culture systems have been shown to significantly reduce turnaround times for microbial identification, improving efficacy and accuracy of microbial tests [67, 68]. In hematology, automated verification systems now confirm up to 99.5% of routine results without human intervention, enabling laboratory personnel to focus on critical or pathological cases [69]. Even the pre-analytical phase has undergone automation in high-throughput laboratories. Institutions such as the Mayo Clinic, which process 12,000–15,000 samples per day, now use robotic systems for specimen reception, sorting, and scanning, optimizing human resource allocation and reducing errors due to sample mishandling [70]. These examples illustrate how automation has allowed laboratory medicine to “do more and do better,” with measurable improvements in reproducibility, throughput, and safety. By contrast, the field of andrology continues to rely heavily on subjective, microscope-based assessments of semen parameters, an approach that remains largely unchanged since the 17th century.

The integration of novel, advanced semen analysis techniques presents also significant challenges related to cost and accessibility, particularly in resource-limited healthcare settings. To address these limitations, some simplified sperm testing devices, such as YO^®^, SEEM^®^, and ExSeed^®^, have been developed. These smartphone-based tools could offer an affordable and user-friendly alternative, with a cost-effectiveness and ease of use that make them particularly promising and suitable for initial assessments in low- and middle-income countries, thereby improving access to male infertility diagnostics [71]. However, their widespread clinical application is still far from being accepted and validated through proper analyses. The experience of other medical disciplines underscores the potential benefits of adopting advanced technologies in andrology. Asmentione above, automation in clinical microbiology and hematology has led to significant improvements in diagnostic accuracy and workflow efficiency. However, in andrology, the transition towards such innovations is impeded by economic constraints, limited availability of specialized equipment, necessity for specialized training, and the resistance to change of andrologists/seminologists. Overcoming these barriers is essential to enhance the standardization and accessibility of semen analysis, ensuring equitable healthcare delivery for all individuals facing male infertility challenges.

## Future perspectives: urgent need for true innovation

Conventional semen analysis could categorize infertile men into specific subgroups, which is highly valuable in research settings, yet clinically not very useful. Considering all the challenges inherent to semen analysis described, the need for Andrologists and Reproductive Clinicians to apply new tools in clinical practice to diagnose male factor is increasingly urgent. The application of artificial intelligence (AI) could be the first easy step, as fashionable and powerful as it is, holding great potential, from enhancing diagnostics to optimizing treatment approaches. But it would be too easy to say that integrating AI tools into clinical practice, would solve the problem. AI presents challenges related to accuracy, data security, and ethical considerations, all of which require careful oversight from the medical community. Recently, several researches explored various AI methodologies, including deep learning and machine learning, to refine semen testing, such as innovative AI-powered devices (i.e. the LensHooke X1 PRO Semen Quality Analyzer). Recent studies have shown that AI can outperform manual semen analysis in specific parameters. For example, a systematic mapping review reported that deep learning models reached an accuracy of nearly 90% in motility classification and an AUC of ~ 0.89 for morphology assessment, demonstrating higher reproducibility compared to manual evaluation [72]. Furthermore, AI-based detection tools have been tested in severe oligozoospermia or non-obstructive azoospermia, showing the ability to identify rare spermatozoa in milliseconds with a precision comparable to, or even exceeding, that of experienced embryologists [73]. These findings suggest that parameters such as motility, morphology, and even rare sperm detection may be particularly well-suited for AI-driven analysis, thereby offering a concrete glimpse into how such tools could complement and enhance current clinical practice. However, while these AI applications show great promise, further studies are still needed before they can be adopted and integrated into routine clinical practice. Thus, the integration of AI in male fertility assessment is still in its infancy, with limited implementation [74]. In the near future, AI-based systems may support clinicians by providing real-time interpretation of complex semen parameters, integrating data from genetic, proteomic, metabolomic and hormonal analyses to identify underlying causes of infertility that are often missed by traditional testing. When embedded within a multidisciplinary approach, these technologies may not only enhance diagnostic accuracy but also streamline clinical workflows, reduce inter-observer variability, and ultimately improve patient counseling and reproductive success rates. On the other hand, with the limitations of the data available from the current methods and technologies, it is possible, even probable, that AI tools will not advance much our diagnostic power (garbage in = garbage out), something that should be considered and prompt us to design more visionary advancements in this field.

On a broader level, in addition to conventional semen parameters, the evaluation of hormones of the hypothalamic-pituitary-gonadal axis is critical for understanding testicular function and spermatogenesis. Abnormalities in this hormonal axis could indicate primary or secondary testicular dysfunction and guide the selection of further diagnostic tests or therapeutic interventions. Importantly, integrating hormonal data with advanced imaging, genetic testing, and emerging AI-driven semen analysis platforms could enhance the interpretation of male reproductive potential. By “feeding” endocrine profiles into AI-based algorithms, alongside morphological, motility, and molecular sperm parameters, it may become possible to decipher the complex, multidimensional “code” of testicular function, potentially improving diagnostic precision and enabling a more personalized approach to male infertility evaluation.

Therefore, we propose that, to improve our ability to really understand what’s behind an individual man’s infertility and, perhaps, general health status, we should have the courage of thinking wide and moving away from the centrality of semen analysis. After four centuries we are still counting sperm. Alternative diagnostic approaches, which could provide meaningful insights into what is happening within the testis, beyond traditional semen analysis, are needed. Other fields of science offer interesting examples of how technology and a multidisciplinary approach, can make true advancements in fields where they were deemed to be impossible.

**Fig. 2:**
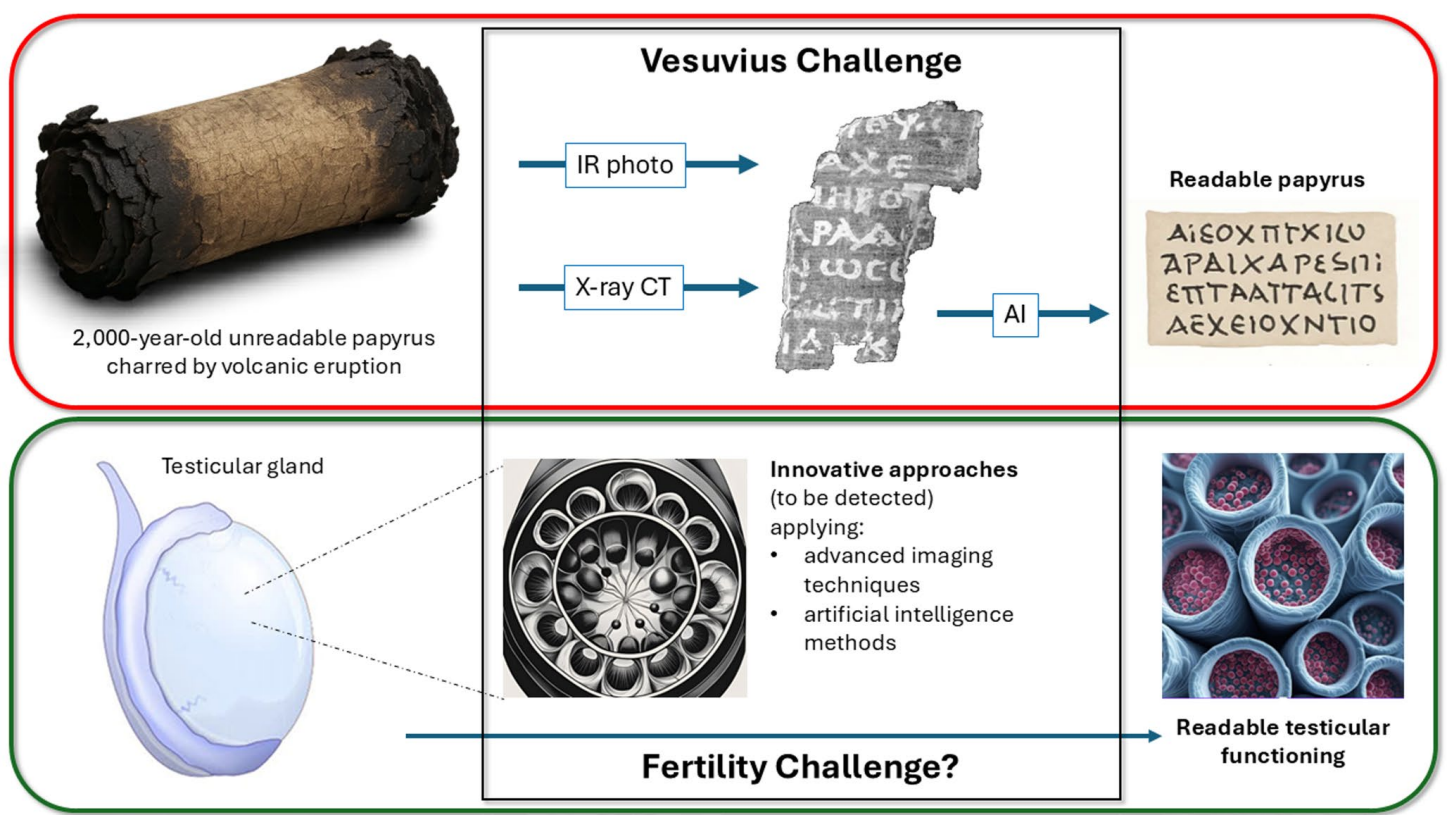
In the red box: A schematic representation of the Vesuvius Challenge. Starting with over 2,000-year-old, unreadable papyrus scrolls charred by a volcanic eruption, artificial intelligence models are developed to detect ink in high-resolution computed tomography (CT) scans of the scrolls, ultimately making the texts readable. In the green box: An illustration of male fertility evaluation. It begins with the testicular gland toward the final desired outcome: describing the testicular functiong. Could innovative strategies, such as those used in the Vesuvius Challenge, be applied here to better predict the final outcome?

One such example coming from archeology is the “Vesuvius Challenge” [75], a multidisciplinary initiative combining high-resolution X-ray phase-contrast tomography and machine learning to virtually unroll and decipher carbonized scrolls from Herculaneum, demonstrating how cutting-edge technologies can extract complex information from fragile, previously unreadable materials [76, 77]. In less than two years, this initiative enabled researchers to reconstruct coherent Greek texts from scrolls that were charred by the eruption of Vesuvius (79 a.C.), and remained sealed and illegible for nearly two millennia. Whith new, cutting-edge technologiers, a multidisciplinary approach, the fantasy of creative young scientists and proper competitive incentives, ancient texts can now be interpreted without physically unwrapping them [78] (Fig. 2). In contrast, we are still unable to non-invasively interrogate the functional state of the testis with comparable precision. This comparison highlights a critical disparity: other scientific domains are rapidly evolving by embracing automation, imaging, and artificial intelligence, while andrology remains confined to outdated paradigms. A shift toward modern, integrative, and technology-driven diagnostics is urgently needed. Thus, looking ahead, we propose a conceptual and methodological shift, moving away from conventional semen analysis. Only by rethinking the diagnostic paradigm at its foundation can andrology catch up with the pace of technological advancement seen in other biomedical fields.

Starting from these perspectives, a pragmatic roadmap can help guide the integration of new technologies into male infertility assessment. In the short term, andrologists and reproductive clinicians should prioritize the implementation of advanced semen analysis tools, from sDF testing to AI-assisted, possibly automated semen analysis assessment. Standardization of protocols, multicenter validation studies, and cost-effectiveness analyses are essential to facilitate broader adoption. Concurrently, research efforts should focus on developing non-invasive, testis-specific diagnostic approaches, leveraging imaging, endocrinology, proteomics, and metabolomics to complement traditional semen analysis. Multidisciplinary collaborations, inspired by successful paradigms in other scientific fields, will be critical to accelerate innovation and translation. By combining near-term improvements with long-term visionary strategies, the field of andrology can move toward a more precise, data-driven, and clinically actionable understanding of male infertility.

## Data Availability

No datasets were generated or analysed during the current study.
